# Hunting promotes sexual conflict in brown bears

**DOI:** 10.1111/1365-2656.12576

**Published:** 2016-08-30

**Authors:** Jacinthe Gosselin, Martin Leclerc, Andreas Zedrosser, Sam M. J. G. Steyaert, Jon E. Swenson, Fanie Pelletier

**Affiliations:** ^1^ Département de BiologieUniversité de Sherbrooke2500 boulevard de l'UniversitéSherbrookeQCJ1K 2R1Canada; ^2^ Department of Environmental and Health StudiesTelemark University CollegeBøNO‐3800Norway; ^3^ Institute of Wildlife Biology and Game ManagementUniversity of Natural Resources and Life SciencesViennaA‐1180Austria; ^4^ Department of Ecology and Natural Resource ManagementNorwegian University of Life SciencesÅsNO‐1432Norway; ^5^ Norwegian Institute for Nature ResearchTrondheimNO‐7485Norway

**Keywords:** cub survival, hunting, male reproductive strategy, Scandinavia, sexually selected infanticide, social restructuration, *Ursus arctos*

## Abstract

1. The removal of individuals through hunting can destabilize social structure, potentially affecting population dynamics. Although previous studies have shown that hunting can indirectly reduce juvenile survival through increased sexually selected infanticide (SSI), very little is known about the spatiotemporal effects of male hunting on juvenile survival.
2. Using detailed individual monitoring of a hunted population of brown bears (*Ursus arctos*) in Sweden (1991–2011), we assessed the spatiotemporal effect of male removal on cub survival.
3. We modelled cub survival before, during and after the mating season. We used three proxies to evaluate spatial and temporal variation in male turnover; distance and timing of the closest male killed and number of males that died around a female's home range centre.
4. Male removal decreased cub survival only during the mating season, as expected in seasonal breeders with SSI. Cub survival increased with distance to the closest male killed within the previous 1·5 years, and it was lower when the closest male killed was removed 1·5 instead of 0·5 year earlier. We did not detect an effect of the number of males killed.
5. Our results support the hypothesis that social restructuring due to hunting can reduce recruitment and suggest that the distribution of the male deaths might be more important than the overall number of males that die. As the removal of individuals through hunting is typically not homogenously distributed across the landscape, spatial heterogeneity in hunting pressure may cause source–sink dynamics, with lower recruitment in areas of high human‐induced mortality.

## Introduction

Human exploitation affects wild vertebrates globally (Milner, Nilsen & Andreassen 2007; Allendorf & Hard 2009) and is considered one of the greatest evolutionary pressures on wildlife (Darimont *et al*. 2009). Large vertebrates are typically harvested for sport hunting, subsistence, or population management (Festa‐Bianchet 2003). Human‐induced mortality in these species generally increases mortality rates in age and sex classes that typically show high natural survival rates (Ginsberg & Milner‐Gulland 1994; Langvatn & Loison 1999; Bonenfant *et al*. 2009). Although several studies have documented the direct demographic consequences of hunting on wild populations, fewer have explored its potential indirect effects (Milner, Nilsen & Andreassen 2007). Indirect effects of hunting often occur through the removal of individuals of specific sex or age classes, mostly through size‐selective hunting, and can destabilize social structures (reviewed in Milner, Nilsen & Andreassen 2007), with negative consequences, such as loss of social knowledge (McComb *et al*. 2001), changes in operational sex ratio (Milner‐Gulland *et al*. 2003) and sexually selected infanticide (SSI) (Swenson *et al*. 1997; Loveridge *et al*. 2007). Understanding the extent of the ecological consequences of hunting is critical when developing sustainable management plans.

Sexually selected infanticide occurs when competition between members of one sex for the reproductive investment of the other sex makes it advantageous for an individual, usually a male, to kill another individual's dependent offspring in order to gain reproductive opportunities (Hrdy 1979). SSI is adaptive when it is directed at young unlikely to be direct descendants of the male (Hrdy 1979). It has been suggested that males assess their paternity through mating history and tend not to kill dependent young (hereafter referred to as juveniles) of females they have mated with (Soltis *et al*. 2000). Therefore, males encountering unfamiliar females with juveniles (hereafter referred to as male turnover) will have a higher probability of perpetrating infanticide. Male turnover has been shown to increase SSI (Swenson *et al*. 1997; Agrell, Wolff & Ylönen 1998; Andreassen & Gundersen 2006), potentially exacerbating the effects of hunting on population dynamics by increasing juvenile mortality after an adult male has been killed (Wielgus *et al*. 2013; Gosselin *et al*. 2015).

The impact of hunting and SSI on juvenile survival may be scale‐dependent and vary temporally. SSI is only adaptive if the male can increase its reproductive opportunities, typically by shortening the interval until the female's next oestrus (Hrdy 1979). Therefore, in seasonal breeders, where females can only be receptive during a short period of the year, SSI is only expected to occur during the mating season and in species where females have the ability to enter oestrus again shortly after losing their young (Hrdy 1979; Steyaert, Swenson & Zedrosser 2014). Juvenile survival should vary spatially, as hunting pressure is often not evenly distributed across the landscape (Lebel *et al*. 2012; Steyaert *et al*. 2016). A female whose home range is near the site where a male has been killed should be more likely to suffer SSI than a female further away. Accordingly, increasing the number of males killed near a female's home range may increase the risk of SSI by opening more space to unfamiliar males. Previous studies have reported that hunting can lead to home range shifts and takeovers in carnivores (Loveridge *et al*. 2007; Maletzke *et al*. 2014). To assess the spatiotemporal effects of male removal on female fitness, however, one needs long‐term detailed monitoring of harvested population with detailed information on female reproduction, offspring mortalities at different times of the year and spatial information on male harvest sites.

Here we evaluated the spatiotemporal effects of hunting adult males on juvenile survival using a long‐term study of marked brown bears (*Ursus arctos* L.), a seasonal breeder, in Scandinavia. Brown bears are solitary non‐territorial animals, and most interindividual interactions occur during the mating season (Dahle & Swenson 2003c; Bellemain, Swenson & Taberlet 2006). Home ranges overlap both intersexually and intrasexually; home range of males are larger (median of 1055 km² in our population) and overlap with several female home ranges (median of 217 km² in our population) (McLoughlin, Ferguson & Messier 2000; Dahle & Swenson 2003b). On a local scale, males adjust their home range size according to population density (Dahle & Swenson 2003b). Home ranges of males only overlap partially, and it is likely than when a male dies, the surrounding adult males will move, adjust or expend their home range in the following years to take advantage of the newly available space (Loveridge *et al*. 2007; Maletzke *et al*. 2014). Due to these home range adjustments, surrounding males may encounter unfamiliar females in the new area they are using. Immigrant males may also take over the newly available home range; however, immigrant males are likely to be young dispersing males, who are less likely to successfully commit infanticide than established older and larger males, as females actively defend their young (Hessing & Aumiller 1994; Støen *et al*. 2006). In this population, most young (95%) are weaned as yearlings and are therefore only dependent during their first year of life (Dahle & Swenson 2003a). A large proportion of litters suffer from partial (17·7%) or total (26·2%) mortality (Gonzalez *et al*. 2012). Approximately 80% of the mortality of cubs of the year (hereafter referred to as cubs) occurs during the mating season (mid‐May to mid‐July, see Fig. S1, Supporting Information), and all causes of death that could be assessed during this period were due to male infanticide (Steyaert 2012; Gosselin *et al*. 2015). Harvest of adult males has been shown to reduce cub survival (Swenson *et al*. 1997, 2001; Zedrosser *et al*. 2009), but we do not know whether the number of bears killed, or their location, affects SSI. We predicted (P1) that increased male turnover would decrease cub survival only during the mating season, (P2) a positive relationship between cub survival and the distance to the closest killed male, and (P3) a negative relationship between cub survival and the number of males killed near a female's home range. We also tested whether the timing of the kill (0·5 or 1·5 years earlier) affected cub survival.

## Materials and methods

The study area was located in south‐central Sweden (61°N, 15°E). Approximately 80% of females and 50% of males of the study population were fitted with VHF radio‐transmitters (models 500 and IMP/40/L HC, Telonics Inc., Mesa, AZ, USA) or GPS‐GMS transmitters (GPS Plus; Vectronic Aerospace GmbH^®^, Berlin, Germany). For further information on capture and handling of bears, see Arnemo, Evans & Fahlman (2011) and Zedrosser *et al*. (2007b). To ascertain timing of cub loss, females with cubs were observed from the ground or from a helicopter at least three times; at den emergence before the breeding season (early May), after the breeding season (mid‐July) and in autumn before denning (late September, early October). Using these censuses, we assessed cub survival before (den emergence to mid‐May), during (mid‐May to mid‐July) and after the mating season (mid‐July to November). The mating season was defined from observation of adult pairs in our study area (Dahle & Swenson 2003a; Steyaert 2012; see Fig. S1).

### Male Turnover

There is a fall bear hunting season in Sweden. Successful bear hunters are required by regulation to provide authorities with the location of the kill, sex of the bear and a tooth for age determination (see Bischof *et al*. 2008 for details). We also have information on damage‐control kills and accidental deaths. We used all known records of mortality of adult male bears (≥3 years old, age of sexual maturity; Zedrosser *et al*. 2007a) to ascertain male turnover, as the impact of a male's death on social structure should be the same regardless of the cause of death. We are confident that we have records of almost all adult male deaths in our study area, because it is legally required to report any bear killed or found dead, regardless of cause of death (including hunting, management removal and accidents), to the appropriate authorities. Illegal kills are rare in this area (Swenson *et al*. 2001; Bischof *et al*. 2009), and natural mortality is low (1·9%, based on 104 mortality records of radio‐marked adult males). Hunting accounted for 85·4% of the 254 male mortalities in the database used in our analyses. Other human causes of mortality explained almost all of the remaining mortality. Hunting is additive to natural mortalities in our study area (Bischof *et al*. 2009). For each female with cubs, we extracted data on all known adult male deaths that occurred within 80 km from the centre of her home range. We chose 80 km, because the maximum documented distance between home range centres of a reproductive pair was 76·8 km (Bellemain *et al*. 2006). Females' home range centres were calculated from the arithmetic centre of annual locations (mean of 57 locations per female per year) for the year their litters were born. Females' home ranges are relatively stable from one year to the next, with home range centroids moving by 1·6 km per year on average. We looked at the impact of past male death on the present cub survival, because male turnover does not occur immediately after male removal (Swenson *et al*. 1997). Therefore, we used data on kill sites for all males in the previous 1·5 years, as it has previously been shown that cub survival is lower when at least one male had been killed in the same area 0·5, and especially 1·5, years earlier (Swenson *et al*. 1997). For each female with cubs in a given year, we calculated the distance between her home range centre and the locations of every male that died during the previous 1·5 years. We used three proxies to evaluate variation in male turnover; distance and timing (0·5 or 1·5 years earlier) of the closest male killed and number of males that died around a female's home range centre.

### Control Covariates

To account for other factors likely to affect cub survival, we included two environmental factors; an annual food index (Zedrosser, Dahle & Swenson 2006) and a population density index, that is an approximation of the number of bears within 1000 km² around the home range centre of a female (Zedrosser, Dahle & Swenson 2006). Covariates describing maternal characteristics were parity (primiparous or multiparous) and female age, their interaction and litter size at first observation after den emergence (litters of 1 cub *n *=* *29, 2 cubs *n *=* *80, 3 cubs *n *=* *77, and 4 cubs *n *=* *7).

### Statistical Analyses

As all covariates were common to a litter, and because survival of cubs within a litter is likely not independent, we modelled cub survival within a litter. We defined cub survival within a litter as the ratio of the number of surviving cubs in relation to the number of cubs in a litter known to be alive at the beginning of each time step: before (after den emergence), during and after the mating season. Analyses were performed on data from 193 litters of 68 females for a total of 448 cubs. We first evaluated if cub survival (dependent covariate) differed before, during and after the mating season, using a generalized linear mixed model with binomial error distribution and Year and Female ID as random intercepts. All subsequent analyses were conducted separately by period.

We did not know *a priori* if the relationship between the distance to the closest male killed and cub survival was continuous or discontinuous, as there might be a threshold effect. Therefore, we performed a preliminary piecewise regression (Crawley 2007). For each period, we compared the complete model (including environmental factors, maternal characteristics and male turnover) with distance to the closest male killed as a continuous or as a discontinuous covariate, with different break points ranging from 10 to 60 km by increments of 5 km (Table S1). For each period, distance to the closest male was selected as continuous or discontinuous, based on the Akaike information criterion corrected for small sample sizes (AICc).

We modelled cub survival using generalized linear mixed models. We evaluated eight candidate models for each period (Table 1). All candidate models were tested with Year and Female ID as random intercepts and fixed effects were based on combinations of the three groups of covariates: environmental factors, maternal characteristics and male turnover. As all tested models were nested, we selected the model with the fewest parameters within ΔAICc <2 of the top model (Arnold 2010). For periods where distance to the closest killed male was retained, we further assessed the effect of the number of males killed and the timing of the closest kill (0·5 or 1·5 years before) on cub survival. All analyses were performed with r 3.1.1 (R Core Team 2014).

**Table 1:** Candidate models tested to explain litter survival before (*n *=* *193), during (*n *=* *185) and after (*n *=* *125) the mating season in brown bears in Sweden during 1991–2011. The variables Year and Female ID were included as random intercepts in all models

Model	Covariates
1	None
2	Food index^a^ + Population density^a^
3	Age of female + Primiparity of female^b^ + Litter size + Age of female × Primiparity of female^b^
4	Distance of the closest killed male (km)^c^
5	Model 2 + Model 3
6	Model 2 + Model 4
7	Model 3 + Model 4
8	Model 2 + Model 3 + Model 4

a Scaled covariate where mean = 0 and variance = 1.

b Primiparous or multiparous.

c Distance was modelled with a breaking point at 55 and 25 km before and during the mating season, respectively, and was modelled without inflexion point after the mating season (see Fig. S2).

## Results

Between 1991 and 2011, mean cub survival within a litter was 0·945 (0·913:0·977, *n *=* *193) before and 0·949 (0·911 : 0·988, *n *=* *125) after the mating season. It was significantly lower during the mating season [0·632 (0·563 : 0·702), *n *=* *185; *z*‐value >6·94, *P*‐value < 0·001]. Of the 185 litters monitored during the mating season, 57% (106) entire litters survived, 32% (60) entire litters died and 10% (19) experienced partial cub loss. Preliminary analyses showed that distance to the closest male killed had a better fit with breaking points at 55 and 25 km before and during the mating season, respectively, and when it was considered as a continuous covariate after the mating season (Fig. S2).

**Figure 1:**
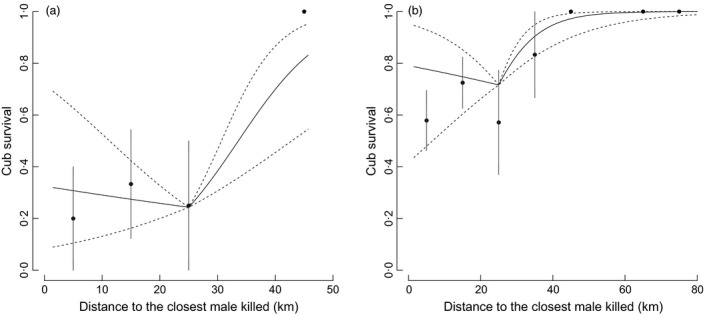
Effects of distance to the closest killed adult male brown bear during the previous 1·5 years on cub survival (*n *=* *185) during the mating season in Sweden during 1991–2011. The predictions are for litter size of 1 cub (panel a) and 2 cubs (panel b; predictions for litter size = 3 or 4 were intermediate). The full and dashed lines represent the predictions of the selected model and its 95% confidence intervals. Dots and vertical lines represent mean cub survival and its 95% confidence interval from raw data segmented every 10 km. Cub survival was calculated by averaging the proportion of cubs surviving per litter, independently of litter size.

Before the mating season, the most parsimonious model of cub survival only included maternal characteristics (Table 2). However, all the confidence intervals of the covariates overlapped with 0 (Table 3). During the mating season, the most parsimonious model included maternal characteristics and male turnover (Table 2). Litter size, parity and their interaction influenced cub survival during the mating season (Table 3). Cubs of older primiparous females had a higher survival than cubs of younger primiparous females, whereas age of the mother had no effect on cub survival for multiparous females (Table 3). Litter size also affected cub survival, with cubs born in litters of 2 or 3 surviving better than cubs born in litters of 1 (Table 3). Regarding male turnover, there was no relationship between distance to the closest killed male and cub survival for distances <25 km (Table 3, Fig. 1), but we found a positive relationship when distances were ≥25 km (Table 3, Fig. 1). Overall, cub survival was lower when the closest killed male was within 25 km than when the closest male was killed farther away (Table 3, Fig. 1). We further tested whether the timing and/or the number of males killed <25 km or ≥25 km explained variation in cub survival. We found no detectable effect of the number of males killed in either analysis (Tables S2 and S3). However, the timing of the closest kill affected cub survival. When the closest male killed was within 25 km of the female, cub survival was 16·6% lower [β = −0·966 (−1·879: −0·052)] when the male was killed 1·5 years earlier compared to when the male had been killed 0·5 year earlier (Table S2). After the mating season, the null model was the most parsimonious (Table 2).

**Table 2:** Model selection diagnostics for the candidate models to explain litter survival before (*n *=* *193), during (*n *=* *185) and after (*n *=* *125) the mating season in brown bears in Sweden during 1991–2011. Models are listed with their Loglikelihood (LL), number of parameters (*K*), difference in Akaike's information criteria (AICc) to the most parsimonious model (∆AICc) and their weight (ω_*i*_). For model description, see Table 1

Model	Before				During				After			
	LL	*K*	∆AICc	ω_*i*_	LL	*K*	∆AICc	ω_*i*_	LL	*K*	∆AICc	ω_*i*_
1	−53·27	3	5·12	0·030	−226·12	3	28·35	0·000	−43·95	3	0·00	0·571
2	−49·66	5	2·25	0·134	−225·79	5	31·90	0·000	−43·56	5	3·41	0·254
3	−44·28	9	0·00	0·384	−216·61	9	22·20	0·000	−40·98	9	6·92	0·018
4	−51·02	5	4·81	0·035	−214·26	5	8·83	0·009	−43·71	4	1·60	0·257
5	−42·61	11	1·13	0·218	−216·30	11	26·05	0·000	−40·58	11	25·59	0·003
6	−47·58	7	2·21	0·127	−212·81	7	25·22	0·004	−43·49	6	5·41	0·038
7	−44·29	11	4·50	0·040	−203·27	11	0·00	0·739	−40·67	10	8·53	0·008
8	−42·23	13	4·95	0·032	−202·08	13	2·18	0·248	−40·45	12	12·61	0·001

**Table 3:** Coefficients (β) and 95% confidence intervals of the covariates in the best supported model to explain brown bear cub survival in Sweden before (*n *=* *193) and during (*n *=* *185) the mating season, respectively (after the mating season, the null model was the most parsimonious)

Covariates	β	95% Confidence intervals	
		Lower limit	Upper Limit
Before the mating season (*n* = 193)			
Intercept	8·588	2·006	15·171
Age	0·183	−0·170	0·536
Primiparity: primiparous	−10·575	−53·251	32·100
Litter size = 2	1·889	−1·409	5·188
Litter size = 3	2·666	−0·732	6·065
Litter size = 4	0·440	−4·047	4·928
Age × Primiparity primiparous	1·352	−7·355	10·058
During the mating season (*n* = 185)			
Intercept	−0·240	−1·979	1·499
Age	−0·087	−0·194	0·021
Primiparity: primiparous	−**8·076**	**−13·487**	**−2·666**
Litter size = 2	**2·039**	**0·699**	**3·379**
Litter size = 3	**1·855**	**0·542**	**3·167**
Litter size = 4	1·399	−0·306	3·103
Distance to the closest killed male (<25 km)	−0·017	−0·080	0·047
Distance to the closest killed male (≥25 km)	**0·132**	**0·069**	**0·196**
Age × Primiparity: primiparous	**1·282**	**0·246**	**2·317**

Numbers in bold represent covariates for which 95% confidence intervals do not overlap 0.

## Discussion

Documenting the indirect effects of hunting yields valuable information that helps to ensure sustainable exploitation of wild populations. Long‐term data sets on marked harvested populations required for documenting such effects are, however, rare (Milner, Nilsen & Andreassen 2007; Clutton‐Brock & Sheldon 2010). In this study, exceptionally detailed information on kill sites and monitoring of female reproductive success allowed us to evaluate the potential indirect spatiotemporal effects of male removal on cub survival. Our analyses revealed three key findings. First, the effect of male removal on cub survival was only apparent during the mating season, in accordance with P1. Second, females with home ranges located closer to sites where at least one male was killed during the previous 1·5 years suffered increased risk of cub loss, in accordance with P2. Third, females with several killed males close to their home range did not suffer an increased risk of cub loss compared to females with only one killed male (contrary to P3), suggesting that even a low rate of harvest can promote SSI.

Cub survival showed a clear temporal pattern, with the lowest survival during the mating season. A study in Alaska also reported higher cub mortality during this period, potentially related to SSI (Gardner, Pamperin & Benson 2014). In contrast, this temporal pattern has not been seen in other North American populations where SSI is thought to be low or absent (Wielgus & Bunnell 1994). In addition to documenting this temporal pattern, we also found that the distance to the closest killed male was a good predictor of cub survival only during the mating season. This temporal effect was expected from the SSI hypothesis, because female brown bears are seasonal breeders and can enter oestrus shortly after losing their young, but almost exclusively during the mating season in Sweden (Fig. S1; Bellemain, Swenson & Taberlet 2006; Steyaert *et al*. 2012; Steyaert, Swenson & Zedrosser 2014). Therefore, SSI would only be beneficial for males during the mating season. The fact that we did not find an indirect effect of hunting on cub survival outside the mating season supports the claim that infanticide is a male reproductive strategy (Swenson *et al*. 1997, 2001; Zedrosser *et al*. 2009). If the non‐parental infanticide observed in our population were a result of exploitation or competition (for example: LeBoeuf & Briggs 1977; Townsend *et al*. 2007), it should occur throughout the year, with probably more cases early in the year, when cubs are younger and more vulnerable (Hrdy 1979). As such, the observed pattern of cub survival differed from that expected in populations of brown bears where there is no or a low rate of SSI and cub survival is high during the mating season (McLellan 2005).

Hunting causes home range shift and takeover in carnivores (Loveridge *et al*. 2007; Maletzke *et al*. 2014). Here, we found that the relationship between distance to the closest killed male and cub survival was non‐continuous, with a threshold at 25 km; survival of litters located within a 25‐km radius of a male killed during the previous 1·5 years was low and stable, but increased gradually at distances >25 km. This suggests that male home range shifts influence female fitness differently according to the spatial scale. It may appear surprising that the distance to the closest male killed had no effect on cub survival within 25 km from the home range centre of a mother and her litter. However, median male home range size in our study area is 1055 km² (corresponding to a 18·3 km radius; Dahle & Swenson 2003b), and males roam over great distances to find females during the mating season, travelling up to 20 km daily (Clevenger, Purroy & Pelton 1990; Dahle & Swenson 2003c). Therefore, any male turnover that occurs within 25 km from a female is likely to increase risk of infanticide. Our results showed that the death of males at a distance ≥25 km was less likely to create turnover affecting a given mother. The closest male killed was within 25 km for 71% of the litters (Fig. S3). A previous study of Scandinavian brown bears has shown that, for litters where paternity could be assigned genetically, fathers were located within 25 km of the female home range centre about 76% of the time and within 40 km 95% of the time (Bellemain *et al*. 2006). This is consistent with the pattern of SSI‐caused cub mortality observed in this study.

We expected that an increase in the number of adult males killed near a female would increase turnover rate and thus reduce cub survival. Surprisingly, however, we found no strong support for this prediction (Table S2). The models that included the number of males were not selected, but were within ΔAICc <2 (Table S2 model D, β = 0·210, CIs = −0·105: 0·526). Thus, it is possible that the number of males killed around a female's home range centre affects cub survival, but that we were unable to detect this small effect given our dataset. However, based on our results, the distance to the closest male killed and the timing of the kill were the main two proxies of male turnover affecting cub survival. As such, we found a binomial response, with the greatest effect being whether or not at least one male had been killed within a 25‐km radius during the previous 1·5 years. This dichotomous relationship between cub survival and male turnover suggests that even low hunting pressure (one male bear killed/1963 km^2^) can reduce cub survival. We therefore suggest that, at the landscape scale, the distribution of male kills might be more important for cub survival than the overall number of males killed. This result may appear to contradict a previous study reporting that increasing overall hunting pressure increased the risk of SSI and reduced cub survival in this population (Gosselin *et al*. 2015). However, increasing hunting quotas and the number of killed males will increase the probability of a female being located in area where a male has been removed. Thus, cub survival is expected to be generally lower in periods of high hunting pressure.

We also found that, during the mating season, cub survival was lower when the closest male killed within 25 km was removed 1·5 years earlier compared to 0·5 year earlier. This supported the hypothesis that male turnover is not an immediate response to male removal (Swenson *et al*. 1997). Infanticide seems to be more likely to occur 1·5 years after a male has died, meaning that it takes over a year for surrounding males to adjust their home ranges after the death of an adjacent male. Although not the main focus of our study, we found that the maternal characteristics had an impact on cub survival. Female parity, age and their interaction influenced cub survival, which might reflect the importance of the mother's experience on the care and protection of cubs (Zedrosser *et al*. 2009). Also the survival of cubs in litters of 2 or 3 was higher than the survival of cubs in litters of 1 and seemed higher than the survival of cubs in litters of 4. Optimum litter size for cub survival could therefore be intermediate, which might reflect a trade‐off between vulnerability to SSI, where being more than one cub in a litter provides protection through a dilution effect, and the competition for resources that lowers cub survival in larger litters (Gonzalez *et al*. 2012). However, other factors may influence the effect of litter size on cub survival, mothers with larger litters, for example, may provide more protection against a potentially infanticidal male, as their investment is larger (Maestripieri & Alleva 1991; Koskela *et al*. 2000). Moreover, the estimate for litter size of 4 should be interpreted with reservation due to low sample size (*n *=* *7).

Other studies have shown how the removal of one or a few specific individuals (through harvest or poaching) can destabilize social structure and, in some cases, have drastic consequences on harvested populations. For example, hunting has been shown to promote SSI in African lions (*Panthera leo* L.), leopards (*Panthera pardus* L.) and cougars (*Felis concolor* L.) (Packer *et al*. 2009; Wielgus *et al*. 2013). Thus, one might expect similar effects of male harvest distribution on female vulnerability to SSI for those species. Natural mortality can also affect the social structure of a population. If human‐caused mortality is additive, as in Scandinavian brown bears (Bischof *et al*. 2009), it will exacerbate this effect. Moreover, in most harvested populations, the human‐caused mortality of adults will be greater than their natural mortality (Ginsberg & Milner‐Gulland 1994; Langvatn & Loison 1999; Bonenfant *et al*. 2009). Harvest distribution may also affect the social structure of species without SSI. In African elephants (*Loxodonta africana* Blumenbach) and killer whales (*Orcinus orca* L.), it has been argued that the removal of older and more experienced individuals can affect the social network and population persistence, as group members typically rely on social knowledge from those individuals (McComb *et al*. 2001; Williams & Lusseau 2006). Thus, in species with stable social structure, the spatiotemporal distribution of harvest is likely to affect local population dynamics, and even low harvest rate can impact local population dynamics. Therefore, the assumption that reducing harvest intensity should increase population growth rate might not always hold.

Our research adds to a growing number of studies documenting the potential indirect effects of hunting on wild populations. As hunting and human‐caused mortalities are usually not distributed homogenously across the landscape (Grilo, Bissonette & Santos‐Reis 2009; Steyaert *et al*. 2016), it is likely to influence the population's local spatial dynamics. Thus, we suggest that spatial heterogeneity in hunting pressure could result in a source–sink dynamics, with zones of high human‐induced mortality and lower recruitment being sinks and contributing less to population growth (Novaro, Funes & Walker 2005).

## Data accessibility

Data will be fully available 3 years after publication to allow early stage PhD student and postdocs to have priority access to the dataset. In the interim period, we will make the data available upon request to anyone who wishes to collaborate with us or repeat our analysis. Data available from the Dryad Digital Repository http://dx.doi.org/10.5061/dryad.tc2cb (Gosselin *et al*. 2016).

## Supporting information


**Fig. S1.** Infanticide cases and attempts in relation to the intensity of the mating season.
**Fig. S2.** AICc of piecewise regression models.
**Fig. S3.** Frequency of the distance to the closest male killed.
**Table S1.** Candidate models tested to determine the shape of the relationship between distance to the closest killed male and litter survival.
**Table S2.** Candidate models to test the effect of the number and timing of males killed, when distance to the closest killed male was <25 km.
**Table S3.** Candidate models to test the effect of the number and timing of males killed, when distance to the closest killed male was ≥25 km.
