# Supplementary material for: Hunting promotes sexual conflict in brown bears
Source: J Anim Ecol. 2016 Aug 30;86(1):35–42. doi: 10.1111/1365-2656.12576 (PMC5215440; doi:10.1111/1365-2656.12576)

SUPPORTING INFORMATION

Hunting promotes sexual conflict in brown bears.

Jacinthe Gosselin, Martin Leclerc, Andreas Zedrosser, Sam M.J.G. Steyaert,

Jon E. Swenson and Fanie Pelletier

Figure S1 Infanticide cases and attempts in relation to the intensity of the mating season

Table S1 Candidate models tested to determine the shape of the relationship between distance to the closest killed male and litter survival

Figure S2 AICc of piecewise regression models

Table S2 Candidate models to test the effect of the number and timing of males killed, when distance to the closest killed male was < 25 km

Table S3 Candidate models to test the effect of the number and timing of males killed, when distance to the closest killed male was ≥ 25 km

Figure S3 Frequency of the distance to the closest male killed

Figure S1. Infanticide cases and attempts (vertical lines) in relation to the intensity of the mating season (red smoother). Black dots represent specific pairs of GPS marked adult male and female brown bears observed concurrently within 30 m of each other during a specific day in our study area in central Sweden (2008-2011). We scaled the observed number of male-female associations per day between 0 and 1 to calculate a relative 'association intensity', which we consider as a proxy for mating season intensity. Data from the infanticide cases were obtained from Steyaert (2012) and Bellemain, Swenson and Taberlet (2006).

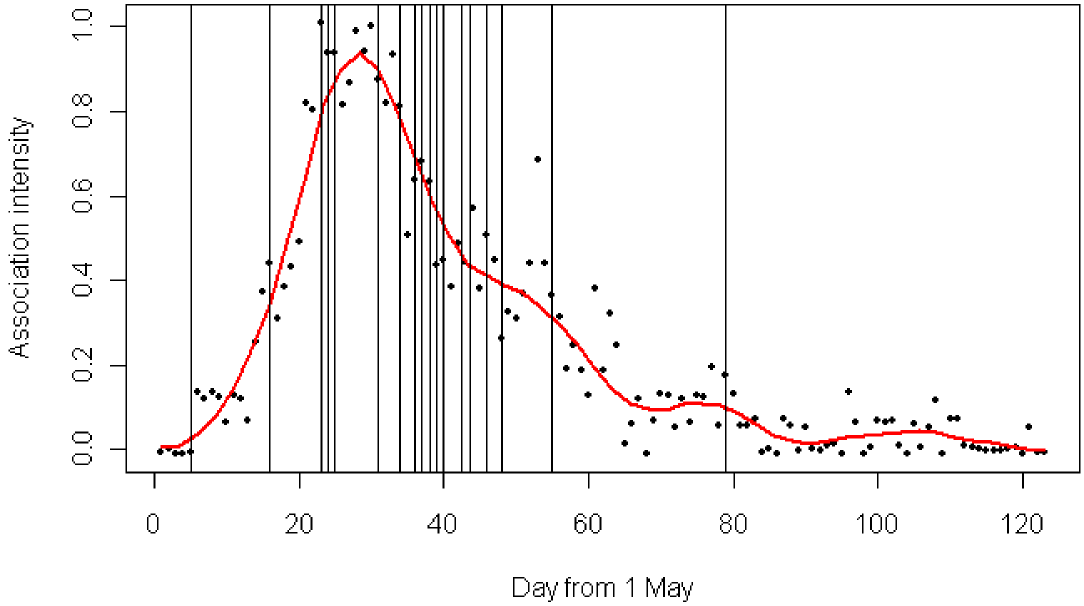

Table S1 Candidate models tested to determine the shape of the relationship (continuous or discontinuous with a breaking point) between distance to the closest killed male and litter before (*n* = 193), during (*n* = 185), and after (*n* = 125) the mating season in brown bears in Sweden during 1991-2011. All models are tested with Year and Female ID as random intercepts.

Model	Covariates
1	Distance of the closest killed male (km) + Food index[a] + Population density[a] + Age of female + Primiparity of female[b] + Litter size + Age of female × Primiparity of female[b]
2	Distance of the closest killed male when <10 km + Distance of the closest killed male when ≥10 km + Covariates model 1
3	Distance of the closest killed male when <15 km + Distance of the closest killed male when ≥15 km + Covariates model 1
4	Distance of the closest killed male when <20 km + Distance of the closest killed male when ≥20 km + Covariates model 1
5	Distance of the closest killed male when <25 km + Distance of the closest killed male when ≥25 km + Covariates model 1
6	Distance of the closest killed male when <30 km + Distance of the closest killed male when ≥30 km + Covariates model 1
7	Distance of the closest killed male when <35 km + Distance of the closest killed male when ≥35 km + Covariates model 1
8	Distance of the closest killed male when <40 km + Distance of the closest killed male when ≥40 km + Covariates model 1
9	Distance of the closest killed male when <45 km + Distance of the closest killed male when ≥45 km + Covariates model 1
25	Distance of the closest killed male when <50 km + Distance of the closest killed male when ≥50 km + Covariates model 1
11	Distance of the closest killed male when <55 km + Distance of the closest killed male when ≥55 km + Covariates model 1
12	Distance of the closest killed male when <60 km + Distance of the closest killed male when ≥60 km + Covariates model 1

[a]Scaled covariate where mean=0 and variance=1; [b]Primiparous or multiparous

Figure S2 Akaike Information Criterion corrected for small sample size (AICc) for piecewise regression models with varying breaking points that explain litter survival (a) before the mating season (*n* = 193), (b) during the mating season (*n* = 185), and (c) after the mating season (*n* = 125) in brown bears in Sweden during 1991-2011. The horizontal dotted line represents AICc value of the model when we did not allow any breaking point (model 1 in Table S1).

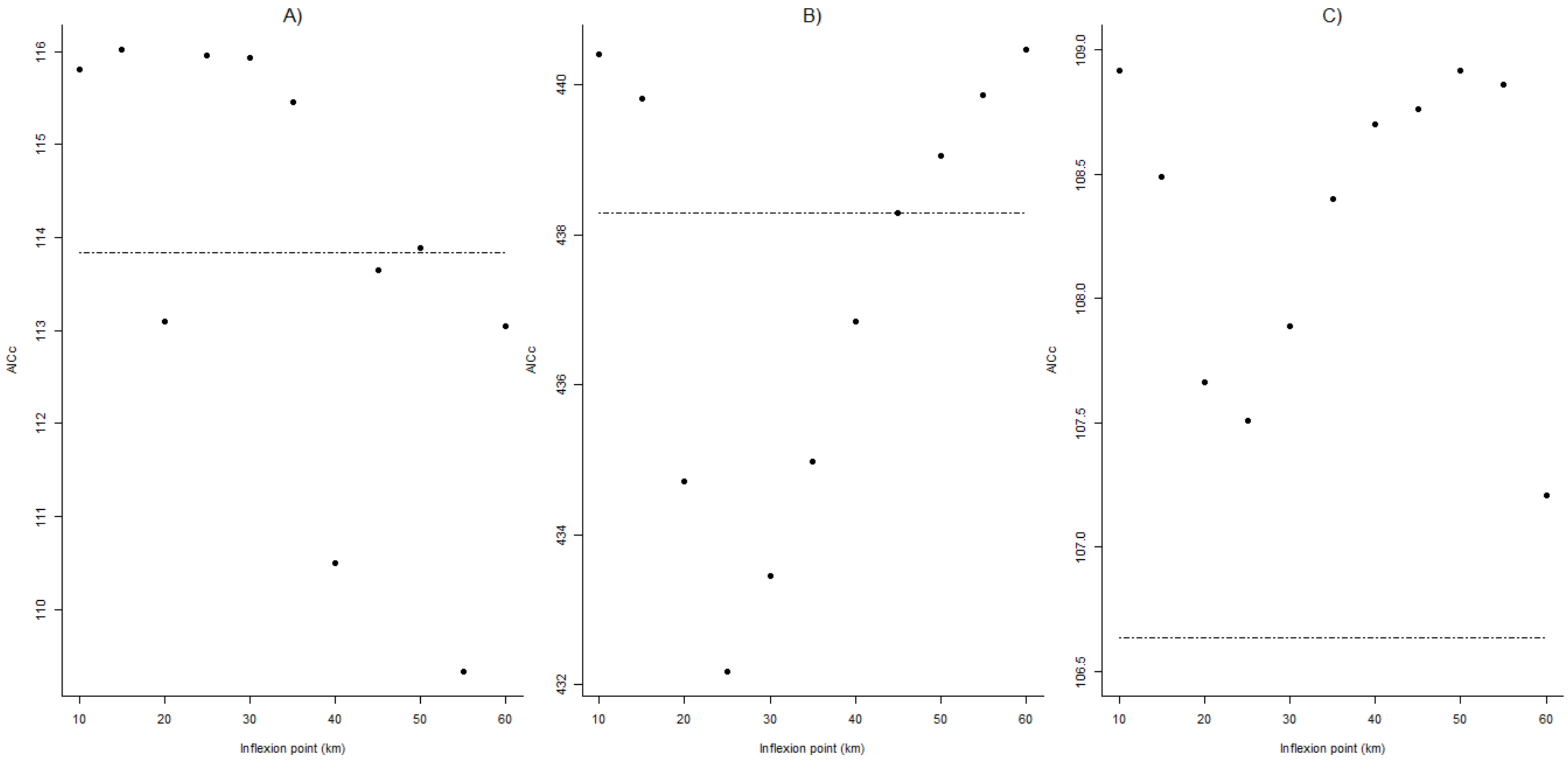

Table S2 Candidate models tested to evaluate the effect of the number and timing of males killed during the hunting season, when distance to the closest killed male was < 25 km, on litter survival (*n* = 131) during the mating season in brown bears in Sweden during 1991-2011. Models are listed with their covariates, LogLikelihood (LL), number of parameters (K), difference in AICc to the most parsimonius model (ΔAICc) and model weight (ω_i).

Model	Covariates included	LL	K	ΔAICc	ω_i
A	Age of female + Primiparity of female[a] + Litter size + Age of female × Primiparity of female[a] + Distance to the closest killed male	-157.042	10	2.44	0.119
B	Model A + Timing[b]	-154.638	11	0.00	0.402
C	Model A + Number of killed males within 25 km	-155.399	11	1.52	0.188
D	Model B + Model C	-153.756	12	0.64	0.292

[a] Primiparous or multiparous, [b] closest male killed 0.5 or 1.5 years earlier

As all tested models were nested, we selected the model with the fewest parameters within ΔAICc <2 of the top model (see methods for details). Therefore, we selected model B. Note that the coefficient of Timing = 1.5 years was -0.966 [-1.879 : -0.052]. Cub survival was 16.6% lower when the timing = 1.5 years earlier instead of 0.5 years earlier (prediction for multiparous female with litter size of 2 cubs and all numeric covariates fixed to the mean). Results obtained are similar to Swenson et al. 1997 *Nature*, 386, 450-451.

Please also note that the effect of the number of male killed (β= 0.210) in model D overlaps with 0 [CIs = -0.105 : 0.526].

Table S3 Candidate models tested to evaluate the effect of the number and timing of adult males killed, when the distance to the closest killed male was ≥ 25 km, on litter survival (*n* = 54) during the mating season in brown bears in Sweden during 1991-2011. Models are listed with their covariates, LogLikelihood (LL), number of parameters (K), difference in AICc to the most parsimonius model (ΔAICc) and model weight (ω_i).

Model	Covariates included	LL	K	ΔAICc	ω_i
A	Age of female + Primiparity of female[a] + Litter size + Age of female × Primiparity of female[a] + Distance to the closest killed male	-32.17	10	0.00	0.946
B	Model A + Timing	-34.16	11	7.10	0.027
C	Model A + Number of killed males within 25 km	-34.91	11	8.59	0.013
D	Model B + Model C	-33.25	12	8.52	0.013

[a]Primiparous or multiparous, [b] closest male killed 0.5 or 1.5 years earlier

Figure S3 Frequency of the distance to the closest adult male killed within the 1.5 previous years of a litter (n = 193) in brown bears in Sweden during 1991-2011.

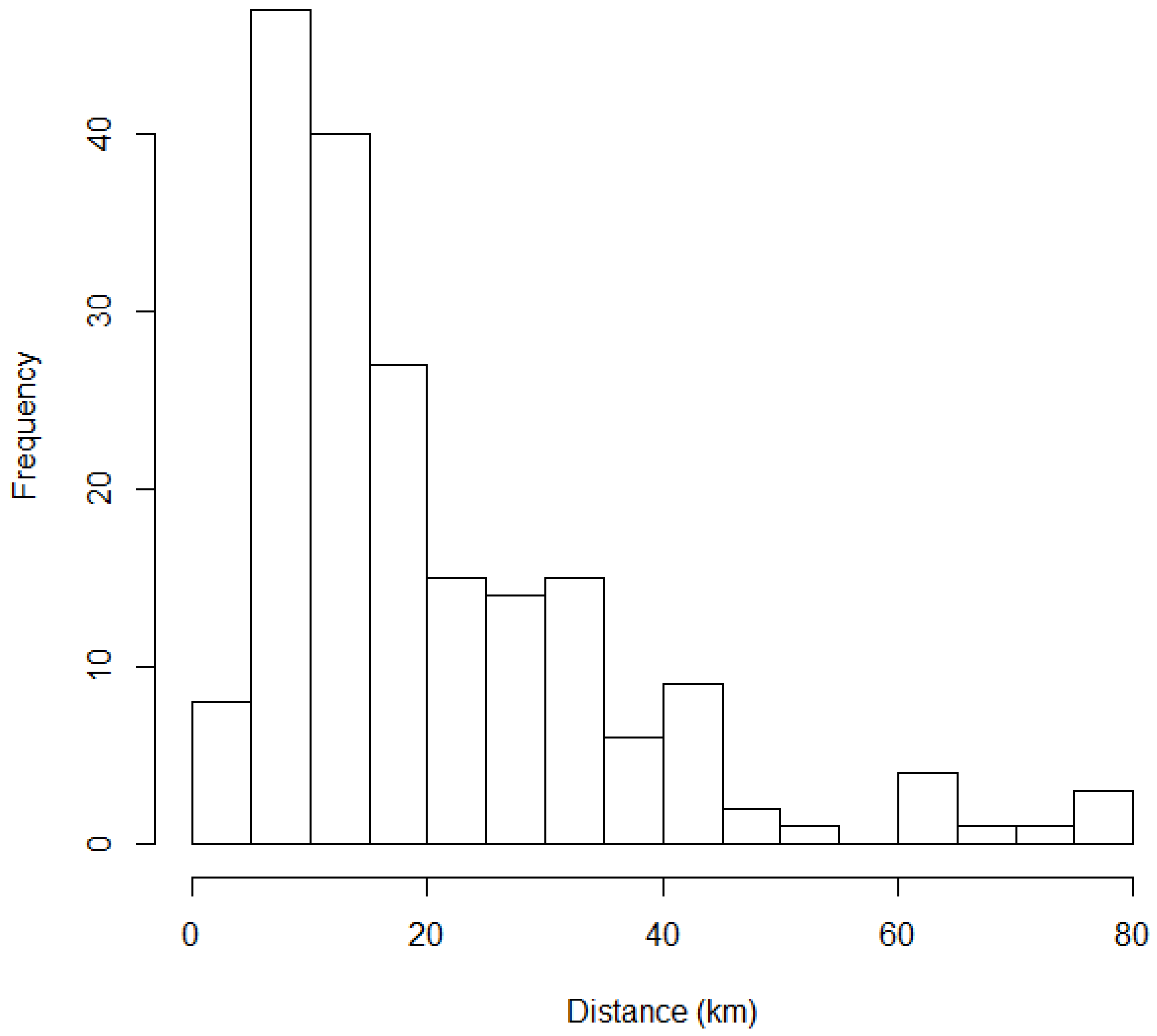

Supplement: Supplementary file 1 — Fig. S1. Infanticide cases and attempts in relation to the intensity of the mating season. Fig. S2. AICc of piecewise regression models. Fig. S3. Frequency of the distance to the closest male killed. Table S1. Candidate models tested to determine the shape of the relationship between distance to the closest killed male and litter survival. Table S2. Candidate models to test the effect of the number and timing of males killed, when distance to the closest killed male was <25 km. Table S3. Candidate models to test the effect of the number and timing of males killed, when distance to the closest killed male was ≥25 km. [file JANE-86-35-s001.pdf]
